# Medical Items and News of Interest to the Physician

**Published:** 1873-01

**Authors:** 


### For the Use of Physicians.

CULLED FROM THE STANDARD MEDICAL JOURNALS OF THE WORLD.

NOTE.—These formulæ are intended only for the regular practitioner of medicine, and should be employed only by him, or under his immediate supervision.

### Incontinence of Urine in Children.

Mr. Holmes Coote recommends for this intractable affection the administration of creosote, in one minim doses,three times daily, combined with assofœtida and rheubarb pill, of each two grains.—*Med. Record.*

### Night Sweats, in Phthisis.

Dr. J. O. Webster, in a letter to the Boston *Med, & Surg. Journal,* says that while surgeon of the National Military Asylum, he had under his care many cases of night sweat, from consumption, and which he almost invariably controlled by oxide of zinc, three grains in a pill, at night.

### Traumatic Tetanus.

Dr. C. Bard reports, in the *Western Lancet,* a case of traumatic tetanus in a girl 15 years of age, successfully treated by the removal of a foreign body, a piece of bone, from the sole of the foot, and by bromide of potassium given in thirty grain doses every hour. Four doses produced a marked improvement, and complete recovery ensued.

### Laxative Mixture.

Prof. Lindsly says the best remedy he has ever tried in habitual constipation, is to take a half drachm of Epsom salts, dissolved in half a pint of water—adding ten drops of Elixir of Vitriol—*one hour before breakfast.* The smaller the dose, the better, provided it will operate. It may be taken for weeks till a cure is effected.

### Asthma.

Dr. Hale, of Kentucky, (*Chicago Med. Times*), uses the following prescription with much success, in asthmetical cases:

℞

Ether sulph. ℥ iss;

Tinct. lobelia, ℥j;

Tinct. stramonii,

Tinct. opii, āā ʒ iv. M.

Sig: Teaspoonful every hour or two until relief is obtained.

**Hemorrhage in Typhoid Fever.**

S. Weed, M. D., of Clyde, N. Y., (*Buffalo Med. & Surg. Jour.*), records five cases of grave hemorrhage from the bowels in typhoid fever, successfully treated by oil of turpentine, in from 20 to 60 drop doses. He is well aware that this remedy has been much used of late in the ordinary dose in certain stages, or to combat certain symptoms of typhoid fever, but not in the hemorrhage occurring during their progress.

---

**Antacid for Heartburn.**

Dr. B. S. Thompson, in the *Am. Practitioner*, recommends this formula :—

℞

Potassæ subcarbonat.;

Spirit lavand. comp., āā ʒ ij;

Sacchar. albi, ʒj;

Tinct. opii.; gtt. xxx;

Aquæ, ℥ iiss. M.

Sig : Tablespoonful as required.

---

**Laxative Pill.**

The following, according to the *Georgia Medical Companion*, is "the favorite laxative pill of a distinguished practitioner:"—

℞

Ext. aloes pulv., oz. ss;
Gambogiæ, dr. j;
Rhei pulv., dr. ss;
Olei cinnamon, gtt. xx.

Make 120 pills.

---

**Spina Bifida.**

Dr. Morton, of Glasgow, (*British Med. Jour.*), records two cases in which spina bifida was cured by injection. He first performed two tentative tappings with a grooved needle, with an interval of four or five days between the two. The tumor was then tapped with a small trocar and canula, allowing about half the contents of the sac to escape, and about a drachm of a solution of iodine in glycerine was injected. The after-treatment consists in dressing with some bland substance, as oil or lard, cleanliness and care, so as to avoid local injury or irritation. Prevention of the complete escape of the cerebro-spinal fluid, he believes to be of the greatest importance; and in the second case he brushed the wound over with collodion, which answered admirably. The presence of this fluid is essential to the functional, if not to the structural integrity of the spinal cord and brain; and if it be allowed to drain away, the child speedily succumbs.

**Anti-Epileptic Mixture.**

Dr. M. Gonzales Echeverria, in an original communication to the Phila. *Medical Times*, recommends the following mixture in epilepsy:—

℞

Potassi bromidi, ʒ iv—ʒ vj;

Liq. potassæ arsenitis, f. ʒ j;

Succi conii, f. ℥ ij—f. ℥ iv;

Aquæ dest., ad f. ℥ vj. M.

Sig: One tablespoonful three times a day, or oftener, as required by the case.

---

**Epilepsy.**

Dr. L. P. Yandall, Jr., (*Am. Practitioner*), reports three cases of the above disease. In the first, a girl 13 yrs. of age, the bromide of potassium alone had been used in full doses for many months without relief. Dr. Y. gave her 26 grains of the bromide three times a day, and one-hundredth of a grain of the sulphate of atropia, night and morning. The attacks soon became less frequent, and in seven months had apparently disappeared. She was directed, however, to continue the medicine twelve months longer.

---

**Bleached Tincture of Iodine.**

It is said that the sulphate of soda will discolor iodine and yet increase its effect. The *Medical Press & Circular* gives a formula for the combination, as follows :

℞

Tinct. iodine;
Glycerine, pure, āā ℥ j;
Sulphite of soda, ʒ j. M.

Rub the salt to a powder in a small mortar, and add the glycerine gradually; then pour in the tincture, and triturate gently until a solution is effected and the mixture assumes an amber color.

---

**Cod-Liver Oil Emulsion.**

That excellent quarterly, *New Remedies*, edited by Professor H. C. Wood, Jr., translates the following from the *Journal de Pharmacie:*

Take of

Tragacanth, 8 parts;

Cold water, 100 parts.

Make into a mucilage.

This by being simply agitated, makes with cod-liver oil in all proportions, a homogeneous mixture, which may be rendered more agreeable by the addition of 4 parts of alcohol with a little oil of bitter almonds and a trace of oil of cinnamon, to every 30 parts.

**Post-Partum Hemorrhage.**

Dr. John I. Miller, of Pleasant Hill, Mo. (*Kansas City Med. Jour.*), publishes two severe cases of post-partum hemorrhage controlled by compression of the abdominal aorta, in which copious injections were employed. He does not claim any originality in the treatment, but, as it is adopted by very few, these cases are recorded to draw attention thereto, and to encourage others to its use, feeling assured that some patients might be saved in this way which otherwise would be lost.

---

**Gonorrhœal Injection.**

Dr. Reynolds, of Louisville Ky. (*Am. Practitioner*), mentions that he has used the following injection with almost uniform success in a number of cases:

℞

Balsam copaiba, ʒ x;

Cryst. carb. soda, ʒ xx;

Water, f. ℥ xxx. M.

To one part of this emulsion add three parts of water, and inject through a catheter carried into the urethra four or five inches, as often as twice or thrice a day. In chronic cases he uses equal parts of water and the emulsion.

---

**Pruritus.**

Mrs. J. I. Brown, Resident Physician Ill. Woman's Hospital, (*Med. Examiner*), speaks highly of sulpho-carbolate of zinc in cases of obstinate pruritus of the vulva. In one particular case, the vulva after being thoroughly bathed with tepid water was washed twice daily with a solution of the sulpho-carbolate of zinc, containing half a drachm to the ounce of distilled water, the parts being allowed to dry without wiping. She was much improved at the end of a week. During the second week the application was made once daily, on retiring at night. At the end of the 3d week the pruritus was wholy relieved.

---

**Syrup of the Lacto-Phosphate of Lime.**

Those practitioners who desire to prepare their own syrup of the lacto-phosphate of lime. in preference to many unreliable so-called preperations, may note the following formula: Take concentrated lactic acid ℥ ij; dilute with ℥ ij of pure water; add of the magma of freshly precipitated phospate of lime, enough to saturate; of orange-flower water ℥ iss. Filter. Then add pure water to make up an ℥ viij mixture, and ℥ xi of white sugar. No heat is to be employed. Each ℥ contains from two to three grains of the dry phosphate of lime. Dose: For an adult, one to two tablespoonsful three or four times a day.

---

**Syphilis.**

The injection of bichlorate of mercury in the treatment of syphilis finds a warm advocate in Dr. Staub, of Strasbourg, (*Traitement de la Syphilis*), who specially recommends the following formula:

℞

Bichloride of mercury, 1.25 gramm.;
Chloride of ammonium, 1.25 gramm.;
Chloride of sodium, 4.15 gramm.;
White of one egg;
Distilled water, 250 gramm. M.

One *centigramme* is injected daily beneath the skin, in two portions, one-half in the morning, the other in the evening.

---

**For Chapped Hands and Lips.**

The following recipe for chapped hands was much approved during the siege of Paris:

Tincture of aloes, 2 to 4 parts;
Glycerine, 30 parts. M.

On retiring to bed, a piece of cloth wet with this is to be applied over the chapped places, and the hands then gloved.

A good recipe for chapped lips is the following:

Spermaceti, four drachms;
White wax, one drachm;
Oil of almonds, two troy ounces;
Glycerine, one ounce.

Melt the spermaceti, wax, and oil together, and when cooling, stir in the glycerine and perfume.

---

**Beriberi.**

An interesting case of beriberi, the symptoms at one time strongly resembling those of aortic aneurism, is detailed in the *Madras Monthly Jour. of Med. Science*, by Dr. S. Williamson, assistant surgeon of the 19th M. N. I. At last advices the patient, private, aged 33 years, was on sick leave to his native village, and doing well. The formula most beneficial to him was the following:

℞

Potas. acet., gr. x;
Tinct. Scillæ, ♏ x;
Spts. nit. æther, ♏ xx;
Liq. ammon. acet., ℥ ij;
Camphor mixture, ℥ j.

Sig: To be taken (one dose), every three hours.

**Small Pox.**

L. C. Churchill, M. D., resident physician to San Francisco Alms House and Pest House, (*West. Lancet*), recommends, from extended experience, the local application of carboilc acid in small-pox. In his cases, from the seventh to the twelfth day of the well-marked eruption, he added a few drops of water to the carbolic acid, rendering it of a thick syrupy consistence, and with a camel's hair pencil applied a single drop to the point of each pustule on the face. In one case every one of these pustules where shriv-elled up, nearly black, and on the 3d day from the application they fell off, leaving no pit; at the lapse of two weeks no pock-mark on the patient's face could be discovered. In another case of vairola confluens, at about the twelfth day of the eruption, the carbollic acid, prepared as before, was applied as a varnish freely to the whole face and ears. The result was most unmistakable. On the face, the eruption dried up with promptness and fell off in large scales; in fact, the cuticle of the whole face came off, leaving the surface smooth and healed up, and would require close inspection to discover whether he had ever had the small-pox. He is in the habit of giving, for about one week, sulphate of soda, internally, in twelve-grain doses, every three hours, to shorten the disease.

---

**Substitute (?) for Vaccination.**

Dr. B. S. Woodworth, of Fort Wayne, one of the oldest and most eminent physicians of Indiana, (*Am. Practitioner*), writes that his theory—which he has held for thirty-four years—is, that pustulation by tartar-emetic is as good a preventive of small-pox as perfect vaccination. He was led to this theory by a series of papers, in 1838–9, published in the Boston *Med. & Surg. Journal*, in which the almost exact resemblance of tartar-emetic pustules and vaccinia was mentioned. This fact, he adds, is familiar to every physician. Not only is the similarity of the two perfect at their full development, but the successive stages are also alike. He asks: Why may not this artificial pustulation be just as good a preventive as vaccination, since the same process has been gone through with, and the same molecular changes? And, certainly there can be nothing more mysterious in the one case than in the other. Some five or six years after he had first entertained this view, an article in the London *Lancet*, by a German author, advanced the same theory, and, he believes, verified by cases. Dr. Woodworth suggests that experiments be made in hospitals, to prove this theory; and closes his communication with these words: "I almost regret that I was ever vaccinated, for I would be willing to run the risk in proving that which I believe true." [Thus, one by one, our fond theories dwindle into insignificance! The wonderful discovery of Jenner must yield to the salt of Both, or the tartar-emetic of Woodworth—which?—ED. BISTOURY.]

---

**Pelvic Peritonitis and Peri-uterine Cellulitis.**

Dr. James L. Brown, of New York, (*Am. Jour. of Med. Sciences*), observes in regard to the treatment of these affections, that the acute stage should be treated on general principles; anodynes, local depletion, fomentations, and sedatives are the chief therapeutic agents. After this stage has passed, the most efficient means of causing the absorption of the products of inflammation are, for both affections, successive fly-blisters over the hypogastrium, and the copious use of the warm vaginal douche every night and morning. The best apparatus for this is a keg that will hold two or three gallons, having an india-rubber tube attached to the stop-cock. This keg being filled with water, as warm as the patient can bear, is placed upon a shelf ten or twelve feet high; the patient being suitably arranged on a lounge underneath, the free end of the tube is introduced high up in the vagina, and the water allowed to flow in a constant stream against the pelvic roof and neck of uterus. The rapid improvement that follows these means, in a majority of cases, is astonishing, even to those who have had much experience in the use of them.

---

**Simple Constipation.**

David Page, M. B., of Kirby-Lonsdale, Eng., says:

For the treatment of simple constipation, resulting from atony of the bowel, the compound liquoice powder is admirably adapted. Whether in simple uncomplicated torpor of the intestines, or in constipation accompanying temporary gastric disorder, the powder, alone or as an auxiliary to appropriate remedies, is preferable to other preparations of its class. In the former, our object is rather to call into play the peristaltic action of the intestine, than to deplete by serous transudation from its walls, and, in the latter, especially, no prudent practitioner would run the risk of aggravating the disordered stomach by the exhibition of purgatives possessed of irritant or drastic properties. The compound liquorice powder is composed of the following constituents, so prepared as to form when incorporated an almost impalpable powder:

℞

Senna leaves, ℥ vj;
Liquorice root, ℥ vj;
Fennel seeds, ℥ iij;
Sulphur, ℥ iij;
Refined sugar, ℥ xviij. M.

The usual dose is a small teaspoonful at bedtime, in water, with which it is easily mixable, forming an agreeable draught.

---

**For Chilblains.**

A French medical journal highly commends the following for this common and annoying affliction:

Tincture of iodine, 1 part;
Labarraque's solution, 2 parts. M.

The affected part is to be well anointed with this, and dried by the fire. It should not be applied where the skin is broken.

The late Mr. Shay says that a perfectly safe and effectual remedy for chilblains is found in the employment of laudanum, taken internally in very small doses, of from two drops for young children, night and morning, up to six or eight for adults. It is in such quantities perfectly harmless, and, as a rule, will effect a cure in the course of four or five days.

The *London Pharmaceutical Journal* gives the following:—

℞

Glycerine, ℥ iij;
Tinct. of arnica, ℥ j;
Spirit of Camphor, ℥ ss;
Rose-water, ʒ j.

To be well rubbed in night and morning.

The following is "Dr. Dewar's Lotion:"—

℞

Sulphurous acid, glycerine, āā ℥ j;
Distilled water, ʒ ij;

℞

Lin. belladona, ʒ ij;

Lin. aconite, ʒ ij;

Acid carbolic, ♏ x;

Collodion flexile, ad ℥ j. M.

Apply with a camel-hair brush.

The above is for unbroken chilblains; if they are broken, the lin. aconite is to be omitted.

**Syphilitic Ulcers of Face.**

Prof. Gross, at a recent clinic at the Jefferson Medical College, as reported in *Phila. Medical Times*, recommended the following treatment:

℞

Potassii iodid., viij.;
Hyd. chlorid. corros., gr. 1-10. M.

To be taken immediately before, or an hour after, each meal. The diet must be light and nutritious, the skin must be kept clean, and avoid taking cold. As a local application, to promote cicatrization:

℞

Liq. hydrarg, nitrat., gr., x.;

Aquæ, f. ℥ j. M.

Use once a day, and have a cloth wet with—

℞

Liq. plumbi subacetas, f. ʒj;

Infus. ulmi, f ℥ viij; to be kept constantly on the part.

---

**Varix.**

The *British Medical Journal* copies an article from *Berliner Klin. Wochenschr.*, upon the good results obtained by German hospital surgeons, from subcutaneous injection of ergotin, in cases of aneurism, and varix of the lower limbs. The first patient upon whom the experiment was made was a man aged sixty, who had suffered for several years from extensive varices of the leg. A solution of two grammes of ergotin was made in seventy-five grammes each of spirits of wine and glycerine, and a quantity containing twelve centigrammes of ergotin was injected at the proximal end of a varix more than two inches long, and as thick as a little finger, lying over the tibia. The injection was repeated every second day. In eight days the varix could not b seen, and in six weeks no trace was left. During the treatment the patient went about as usual. Another varix, of the size of a hazel-nut, lying on the outer side of the calf, was treated in a similar manner, with the same result. At the point where the injection was made there was some hard circumscribed infiltration, which gradually disappeared. Several other patients in the Greifswald Hospital, some of them with very large varices, have been treated with the subcutaneous injection of ergotin with surprisingly good results. Dr. Vogt believes that the ergotin causes contraction of he muscular coat of the arteries, so that the flow of blood into the dilated vessels is hindered; that it also produces contraction of the veins and that the local infiltration following the injection may have some effect by the compression which it exercises.

---

**Cholera Infantum.**

John O'Reilly, M. D., of Louisville, Ky., (*Medical Practitioner*), recommends, in the gastric type, where exhaustion soon occurs, and cerebral symptoms are apt to set in early, calomel and bromide of potassium, with tincture of hyoscyamus, as follows:

℞
Calomel, gr. x.
Pepsin,
Subnit. bismuth, āā gr. iij. M.

Divide into ten parts, of which give one every hour.

℞

Bromide potassium, ʒ ss;

Tinct. hyoscyamus, ʒ ij;

Water, f. ℥j. M.

Sig: A teaspoonful every three or four hours.

The bromide of potassium acts as a brain sedative, the hyoscyamus exerts a general soothing power, while the calomel, by its peculiar purgative action, relieves gastric congestion. In cholera infantum of the intestinal form he prescribes the following:

℞

Acetate of lead, gr. iv;

Glycerine, ʒ j;

Mint-water, ℥ ss;

Tinct. opium, gtt. iij;

Distilled water, ʒ iij. M.

Sig: A teaspoonful every two hours, until the operations are less frequent. This alone frequently relieves the patient; but where the case is of any standing, calomel is generally required, and then he directs the following:

℞
Calomel, gr. iv;
Bicarb. potash, gr. iv;
White sugar, gr. ij. M.

Divide into four parts, and give one morning and evening.

---

The *New Russian Telegraph* states that the construction of a navigable canal to connect the port of Sebastopol with Balaklava has been resolved upon by the Government, at an estimated cost of 14,000,000 roubles. Sebastopol is to be merely a commercial harbor, and Balaklava a war harbor. Their connection is therefore indispensable.

